# Supplementary material for: Eating habits, lifestyle behaviors and stress during the COVID-19 pandemic quarantine among Peruvian adults
Source: PeerJ. 2021 May 11;9:e11431. doi: 10.7717/peerj.11431 (PMC8121059; doi:10.7717/peerj.11431)
Supplement: Supplemental Information 2 [file peerj-09-11431-s002.pdf]

Anexo 1: Consentimiento informado

"ESTILOS DE VIDA EN EL CONTEXTO DE LA CUARENTENA POR LA PANDEMIA DEL COVID-19 EN ADULTOS PERUANOS"

CONSENTIMIENTO INFORMADO

Estimado Señor(a),

Lo/la invitamos a participar en un estudio de investigación con la finalidad de conocer su estado de salud con relación al peso y a los factores conductuales asociados que podrían estar modificándolo, y en particular, cuáles fueron los efectos positivos o negativos de **su cuarentena en sus últimos 30 días**. Su participación es voluntaria. Si usted acepta participar, se le solicitará a continuación, responder una encuesta virtual (puede ser también vía e-mail o WhatsApp si así lo deseara).

BENEFICIOS:

A pesar de que no se beneficiará directamente con el estudio, posteriormente usted y la comunidad médica podrían hacerlo. Se le informará de manera personal y confidencial los resultados que se obtengan de esta encuesta.

Los resultados del estudio proporcionarán información sobre sobre cómo la cuarentena impactó en la vida de los peruanos y en particular, conocer su impacto en el peso y en diversos comportamientos que podrían haber hecho que este se modifique. Con estos resultados se podrían plantear estrategias y/o intervenciones en el futuro, dirigidas a fomentar estrategias que controlen una modificación del peso y limitar los factores de riesgo que pudieran generar y perpetuar un aumento de peso, llevándolo a un estado de sobrepeso y obesidad que lo colocaría en una categoría de riesgo para desarrollar una enfermedad severa o grave del COVID-19.

COSTOS E INCENTIVOS:

Usted no pagará nada por participar en este estudio. Del mismo modo, no recibirá ningún incentivo económico ni de otra índole por participar en este estudio. Únicamente tendrá la satisfacción de colaborar con una investigación enmarcada en el tema de mejoría de tratamiento médico en el ámbito de la obesidad.

RIESGOS E INCOMODIDADES:

No existen riesgos para su salud física, mental ni emocional por participar en este estudio. No se le realizará ningún procedimiento. Ninguna persona ajena al estudio podrá acceder a la información obtenida mediante esta encuesta, ni a los correos electrónicos o mensajes que intercambiemos. La información se almacenará directamente en una base de datos virtual y solo los investigadores tendrán la clave de acceso. La información de contacto (correos electrónicos y teléfonos) que nos proporcione será almacenada en una base de datos y será eliminada una vez concluido el estudio.

PROCEDIMIENTO:

Para contactarlo, hemos tomado sus datos de una encuesta preliminar que se hizo también vía redes sociales (Facebook) en la cuenta profesional de la Dra. Hellen S. Agurto.

Si decide participar en este estudio, usted deberá completar la encuesta, contestándola directamente a través de esta plataforma, por teléfono o vía e-mail. Una vez que concluya el cuestionario, acabará su participación en el estudio, la misma que le tomará un máximo de 10 minutos.

CONFIDENCIALIDAD:

Le podremos garantizar que la información que usted brinde será absolutamente confidencial. Ninguna persona, excepto los investigadores, manejará la información obtenida. La encuesta no es anónima, sin embargo, serán codificadas de tal manera que, para los resultados, el análisis de datos y la publicación del artículo no figurarán nombres ni apellidos.

Usted puede hacer todas las preguntas que desee antes de decidir si desea participar o no, nosotros responderemos gustosamente. Si una vez que usted haya aceptado participar, luego se desanima o ya no desea continuar, puede hacerlo sin ninguna preocupación, igual le brindaremos la información respecto los factores que condicionan un aumento de peso en el periodo de cuarentena debido a la emergencia sanitaria por el COVID-19 (haciéndole llegar una copia de los resultados obtenidos una vez se haya mandado a una revista científica para su publicación).

CONTACTO:

Cualquier duda respecto a esta investigación puede consultar con la Investigador Principal Dra. Hellen S. Agurto al teléfono 94143-5550, quién estará gustosa de contestar cualquier duda al respecto. También puede comunicarse con el Comité Institucional de Ética en Investigación de IMPACTA, Lima, Perú.

Cordialmente,

Dra. Hellen S. Agurto Lescano
Médico Gastroenterólogo y Endoscopista Bariatra.
C.M.P. 29389 – R.N.E. 14803
D.N.I. 02808719
