# Supplementary material for: Eating habits, lifestyle behaviors and stress during the COVID-19 pandemic quarantine among Peruvian adults
Source: PeerJ. 2021 May 11;9:e11431. doi: 10.7717/peerj.11431 (PMC8121059; doi:10.7717/peerj.11431)
Supplement: Supplemental Information 3 [file peerj-09-11431-s003.pdf]

Anexo 2: Instrumentos de recolección de los datos

Se utilizará una encuesta condensada que contiene cinco (05) encuestas independientes, las mismas que recabarán información de los **últimos 30 días previos al término de la cuarentena** en Lima.

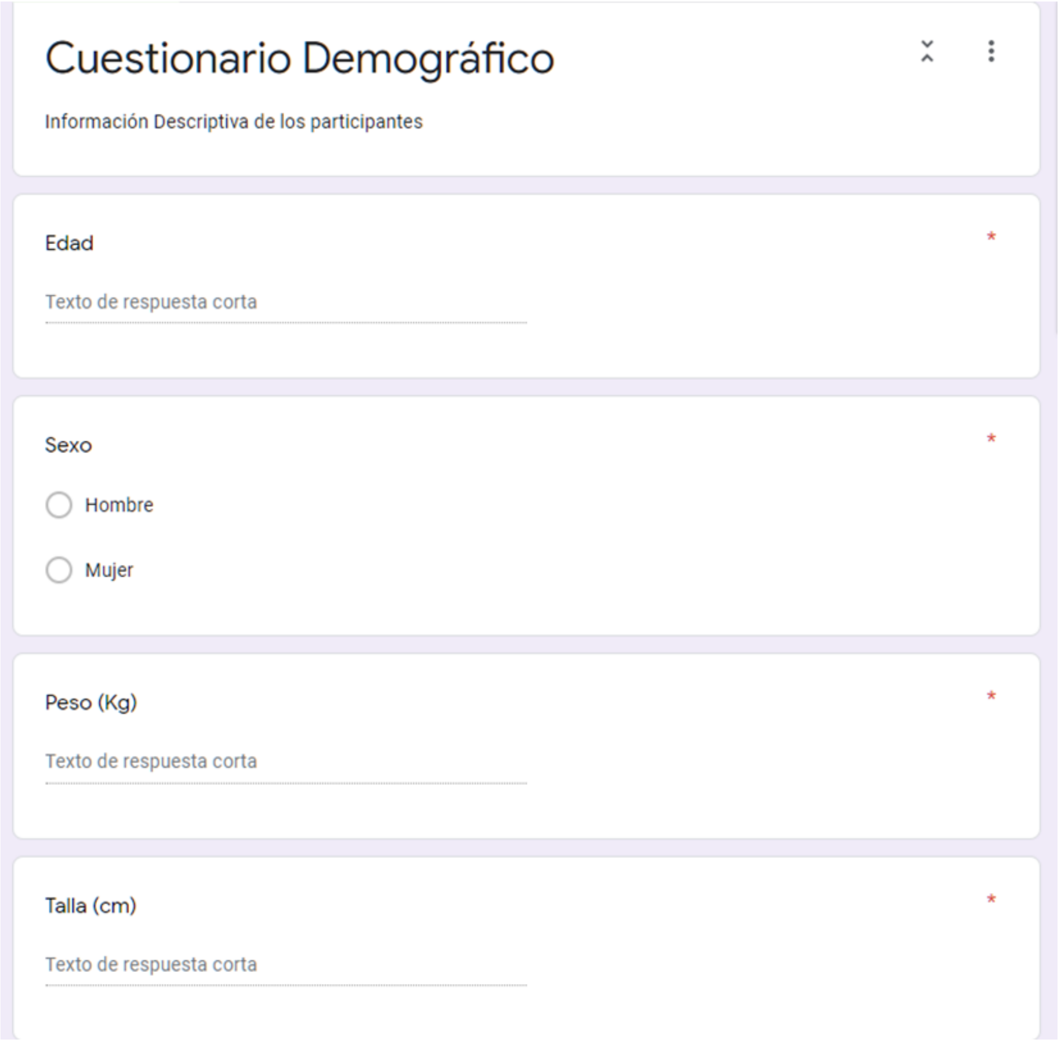

Cuestionario Demográfico

Información Descriptiva de los participantes

Edad *

Texto de respuesta corta

Sexo *

- Hombre
- Mujer

Peso (Kg) *

Texto de respuesta corta

Talla (cm) *

Texto de respuesta corta

Empleo *

Texto de respuesta corta

Estado civil *

- [ ] Soltero(a)
- [ ] Casado(a)
- [ ] Viudo(a)
- [ ] Separado(a)

Tiempo de permanencia en domicilio (horas) *

- ○ 8-12 horas
- ○ 12-16 horas
- ○ 16-20 horas
- ○ 20-24 horas

¿Ha habido algún cambio en su peso? *

- ○ He perdido más de 5 kg
- ○ He perdido entre 2.5 y 5 kg
- ○ He mantenido el peso
- ○ He ganado entre 2.5 y 5 Kg
- ○ He ganado más de 5 kg

¿Con qué precisión cree Ud. que puede reportar su peso? En una escala del 1 al 10 *

1	2	3	4	5	6	7	8	9	10
○	○	○	○	○	○	○	○	○	○

CONDUCTAS ALIMENTARIAS & DEPORTE

Indique las frecuencias de las sgtes. conductas alimentarias y de deporte realiza, según la escala numérica que se presenta a continuación:

¿Come Ud. entre comidas? *

- Nunca
- Una vez al mes
- Dos a tres veces al mes
- Una o dos veces a la semana
- Tres a cuatro veces por semama
- Todos los dias

¿Hace ayunos prolongados? *

- Nunca
- Una vez al mes
- Dos a tres veces al mes
- Una o dos veces a la semana
- Tres a cuatro veces por semama
- Todos los dias

¿Come Ud. más rápido que la mayoría de las personas? *

- Nunca
- Una vez al mes
- Dos a tres veces al mes
- Una o dos veces a la semana
- Tres a cuatro veces por semama
- Todos los dias

¿Come Ud. hasta sentirse incómodo? *

- Nunca
- Una vez al mes
- Dos a tres veces al mes
- Una o dos veces a la semana
- Tres a cuatro veces por semama
- Todos los dias

¿Come Ud. sin sentir hambre físico? *

- Nunca
- Una vez al mes
- Dos a tres veces al mes
- Una o dos veces a la semana
- Tres a cuatro veces por semama
- Todos los dias

¿Come Ud. porque vé comer a los demás? *

- Nunca
- Una vez al mes
- Dos o tres veces al mes
- Una o dos veces a la semana
- Tres a cuatro veces por semama
- Todos los dias

¿Tiene culpa o tristeza despues de comer? *

- Nunca
- Una vez al mes
- Dos a tres veces al mes
- Una o dos veces a la semana
- Tres a cuatro veces por semama
- Todos los dias

¿Se siente estresado por su manera de comer? *

- Nunca
- Una vez al mes
- Dos a tres veces al mes
- Una o dos veces a la semana
- Tres a cuatro veces por semama
- Todos los dias

¿Toma gaseosas? *

- Nunca
- Una vez al mes
- Dos a tres veces al mes
- Una o dos veces a la semana
- Tres a cuatro veces por semana
- Todos los dias

¿Toma jugos procesados (ej: Frugos), malteadas o batidos? *

- Nunca
- Una vez al mes
- Dos a tres veces al mes
- Una o dos veces a la semana
- Tres a cuatro veces por semama
- Todos los dias

¿Toma agua pura? *

- Nunca
- Una vez al mes
- Dos a tres veces al mes
- Una o dos veces a la semana
- Tres a cuatro veces por semama
- Todos los dias

¿Siente Ud. antojos por alimentos dulces? *

- Nunca
- Una vez al mes
- Dos a tres veces al mes
- Una o dos veces a la semana
- Tres a cuatro veces por semama
- Todos los dias

¿Siente Ud. antojos por alimentos salados? *

- Nunca
- Una vez al mes
- Dos a tres veces al mes
- Una o dos veces a la semana
- Tres a cuatro veces por semama
- Todos los dias

¿Siente Ud. antojos por alimentos altos en grasas? *

- Nunca
- Una vez al mes
- Dos a tres veces al mes
- Una o dos veces a la semana
- Tres a cuatro veces por semama
- Todos los dias

¿Toma jugos naturales? *

- Nunca
- Una vez al mes
- Dos a tres veces al mes
- Una o dos veces a la semana
- Tres a cuatro veces por semama
- Todos los dias

¿Deja el plato "vacio" cuando termina de comer? *

- Nunca
- Una vez al mes
- Dos a tres veces al mes
- Una o dos veces a la semana
- Tres a cuatro veces por semama
- Todos los dias

¿En promedio cuántos días a la semana realiza las siguientes comidas? *

	Un dia	Dos días	Tres dias	Cuatro días	Cinco días	Seis días	Siete días
Desayuno	○	○	○	○	○	○	○
Almuerzo	○	○	○	○	○	○	○
Cena	○	○	○	○	○	○	○

¿Cuántas comidas principales tiene por dia? *

1 2 3 4 5

¿Cuánto es el número de "entre comidas" que hace al día? *

0 1 2 3

¿Evita usted algún alimento con intención de cuidar su figura? *

- Si
- No

Si su respuesta anterior fue "Si", díganos ¿a qué alimento se refiere?

Texto de respuesta corta

¿Realiza Ud. ejercicio físico? *

- Sí
- No

Si su respuesta fue "Sí" ¿Qué tipo de ejercicio realiza? (Puede marcar una o más de una)

- Aeróbicos (cardio, steps, etc)
- Anaeróbico (pesas, máquinas, etc)
- Bicicleta estática
- Caminadora
- Elíptica
- Baile (moderno, marinera, ballet, etc)
- Ping-Pong
- Yoga
- Pilates
- Otros

En caso haya respondido OTROS en la respuesta anterior, díganos ¿qué otro ejercicio realiza en casa?

Texto de respuesta corta

Si Ud. SI realiza ejercicio ¿Con qué frecuencia lo realiza? (dias por semana)

1	2	3	4	5	6	7
○	○	○	○	○	○	○

¿Qué tiempo realiza ejercicio? (minutos)

- ○ Menos de 20 minutos
- ○ Entre 20 y 40 minutos
- ○ Entre 40 y 60 minutos
- ○ Más de una hora

Cuestionario de Comportamiento Sedentario

Las preguntas a continuación estan en relación a su actividad en el último mes (30 días):

¿Qué cantidad de tiempo ha permanecido sentado? *

- ○ Mucho menos de lo normal
- ○ Un poco menos de lo normal
- ○ Más o menos igual
- ○ Un poco más de lo normal
- ○ Mucho más de lo normal

¿A qué hora se ha LEVANTADO en promedio en el último mes? *

Hora

¿A qué hora se ha ACOSTADO en promedio en el último mes? *

Hora

En el último mes ¿Cuánto tiempo hizo siesta (minutos)? *

- ◯ 0 min
- ◯ 1-15 min
- ◯ 16-30 min
- ◯ 31-60 min
- ◯ Más de 1 hora

En el último mes ¿Cuánto tiempo -en minutos- estuvo sentado en cada una de las comidas? *

	0 min	1-10 min	11-20 min	21-30 min	31-60 min	Más de 1 h
Desayuno	◯	◯	◯	◯	◯	◯
Almuerzo	◯	◯	◯	◯	◯	◯
Cena	◯	◯	◯	◯	◯	◯

¿Tuvo Ud. una ocupación este último mes (trabajo, estudio o voluntariado)? *

- ◯ Sí
- ◯ No

Si su respuesta anterior fue "Sí" ¿Qué tipo de ocupación tuvo?

- ☐ Trabajo
- ☐ Estudio
- ☐ Voluntariado

¿Cuántos días realizó esta "ocupación" en esta última semana?

1	2	3	4	5	6	7
◯	◯	◯	◯	◯	◯	◯

En el último mes ¿Cuánto tiempo permaneció Ud. sentado mientras se DESPLAZABA hacia y desde su ocupación (en auto, bus o moto). No incluya bicicleta de pedales.

- ◯ 0 min
- ◯ 1-15 min
- ◯ 16-30 min
- ◯ 31-60 min
- ◯ 1-2 h
- ◯ 2-3 h
- ◯ 3-4 h

En este último mes, ¿Cuánto tiempo al dia pasó sentado en promedio mientas REALIZABA SU OCUPACIÓN?

- ◯ 1-30 min
- ◯ 31 min-1 hr
- ◯ 1-2 h
- ◯ 2-3 h
- ◯ 3-4 h
- ◯ 4-5 h
- ◯ 5-6 h
- ◯ 6-7 h
- ◯ 7-8 h
- ◯ Más de 8 h.

En el último mes en promedio, ¿Cuánto tiempo (en minutos) permaneció sentado o echado frente a pantallas? *

	0 min	1-15 min	16-30 ...	30-60 ...	1-2 h	2-3 h	3-4 h	5-6 h	6-7 h	Más d...
Viendo...	○	○	○	○	○	○	○	○	○	○
Usand...	○	○	○	○	○	○	○	○	○	○
Jugan...	○	○	○	○	○	○	○	○	○	○

En el último mes en promedio, ¿Cuánto tiempo (en minutos) permaneció sentado mientras realizaba las siguientes actividades? *

	0 min	1-15 min	16-30 min	30-60 min	1-2 h	2-3 h	3-4 h
Leyendo u...	○	○	○	○	○	○	○
Desarrolla...	○	○	○	○	○	○	○
Cuidando n...	○	○	○	○	○	○	○
Pasatiemp...	○	○	○	○	○	○	○
Escuchand...	○	○	○	○	○	○	○
Otras activi...	○	○	○	○	○	○	○

Si su respuesta fue "Otras actividades", especifiquela.

Texto de respuesta corta

Escala de Stress Auto-percibido

Las siguientes preguntas estan en relación a lo pasado durante el ULTIMO MES (últimos 30 días):

¿Con qué frecuencia ha estado afectado por algo que ha ocurrido inesperadamente? *

- Nunca
- Casi nunca
- De vez en cuando
- A menudo
- Muy a menudo

¿Con qué frecuencia se ha sentido incapaz de controlar las cosas importanes de su vida? *

- Nunca
- Casi nunca
- De vez en cuando
- A menudo
- Muy a menudo

¿Con qué frecuencia se ha sentido nerviso o estresado? *

- Nunca
- Casi nunca
- De vez en cuando
- A menudo
- Muy a menudo

¿Con qué frecuencia ha estado seguro sobre su capacidad de manejar sus problemas personales? *

- Nunca
- Casi nunca
- De vez en cuando
- A menudo
- Muy a menudo

¿Con qué frecuencia ha sentido que las cosas le van bien? *

- Nunca
- Casi nunca
- De vez en cuando
- A menudo
- Muy a menudo

¿Con qué frecuencia ha sentido que no podia afrontar todas las cosas que tenia que hacer? *

- Nunca
- Casi nunca
- De vez en cuando
- A menudo
- Muy a menudo

¿Con qué frecuencia ha podido controlar las dificultades de su vida? *

- Nunca
- Casi nunca
- De vez en cuando
- A menudo
- Muy a menudo

¿Con qué frecuencia se ha sentido que tenia todo bajo control? *

- Nunca
- Casi nunca
- De vez en cuando
- A menudo
- Muy a menudo

¿Con qué frecuencia se ha sentido que las dificultades se acumulan tanto que no puede superarlas? *

- Nunca
- Casi nunca
- De vez en cuando
- A menudo
- Muy a menudo
